# *In vivo* Molecular Imaging of Glutamate Carboxypeptidase II Expression in Re-endothelialisation after Percutaneous Balloon Denudation in a Rat Model

**DOI:** 10.1038/s41598-018-25863-1

**Published:** 2018-05-09

**Authors:** Heike Endepols, Felix M. Mottaghy, Sakine Simsekyilmaz, Jan Bucerius, Felix Vogt, Oliver Winz, Raphael Richarz, Philipp Krapf, Bernd Neumaier, Boris D. Zlatopolskiy, Agnieszka Morgenroth

**Affiliations:** 1 Institute of Radiochemistry and Experimental Molecular Imaging (IREMB), University Hospital of Cologne, 50937 Cologne, Germany; 2 Department of Nuclear Medicine, University Hospital of Cologne, 50937 Cologne, Germany; 3 Department of Nuclear Medicine, University Hospital, RWTH Aachen, 52074 Aachen, Germany; 4 Department of Nuclear Medicine, Maastricht University Medical Centre (MUMC+), 6229 HX Maastricht, The Netherlands; 5 Institute for Molecular Cardiovascular Research, University Hospital, RWTH Aachen, 52074 Aachen, Germany; 6 Cardiovascular Research Institute Maastricht (CARIM), Maastricht University Medical Centre (MUMC+), 6229 HX Maastricht, The Netherlands; 7 Department of Cardiology, Pneumology, Angiology, and Internal Intensive Care Medicine, University Hospital, RWTH Aachen, 52074 Aachen, Germany; 8 Max Planck Institute for Metabolism Research, 50931 Cologne, Germany; 9 Institute for Neuroscience and Medicine (INM-5), Nuclear Chemistry, Research Centre Jülich, 52425 Jülich, Germany

## Abstract

The short- and long-term success of intravascular stents depends on a proper re-endothelialisation after the intervention-induced endothelial denudation. The aim of this study was to evaluate the potential of *in vivo* molecular imaging of glutamate carboxypeptidase II (GCPII; identical with prostate-specific membrane antigen PSMA) expression as a marker of re-endothelialisation. Fifteen Sprague Dawley rats underwent unilateral balloon angioplasty of the common carotid artery (CCA). Positron emission tomography (PET) using the GCPII-targeting tracer [^18^F]DCFPyL was performed after 5–21 days (scan 60–120 min post injection). In two animals, the GCPII inhibitor PMPA (23 mg/kg BW) was added to the tracer solution. After PET, both CCAs were removed, dissected, and immunostained with the GCPII specific antibody YPSMA-1. Difference of GCPII expression between both CCAs was established by PCR analysis. [^18^F]DCFPyL uptake was significantly higher in the ipsilateral compared to the contralateral CCA with an ipsi-/contralateral ratio of 1.67 ± 0.39. PMPA blocked tracer binding. The selective expression of GCPII in endothelial cells of the treated CCA was confirmed by immunohistological staining. PCR analysis verified the site-specific GCPII expression. By using a molecular imaging marker of GCPII expression, we provide the first non-invasive *in vivo* delineation of re-endothelialisation after angioplasty.

## Introduction

Percutaneous vascular intervention (PVI) leads to mechanical endovascular injury with endothelial denudation. A timely re-endothelialisation after stent deployment or balloon angioplasty is essential for safety and efficacy of PVI. The arterial healing involves regrowth of the denuded endothelium from the remaining endothelial cells and uninjured segments proximally and distally of the treated lesion^1,2^. Circulating endothelial progenitor cells play most likely an important role in this process^3^. Current concepts of PVI optimization include approaches to accelerate re-endothelialisation, at the same time trying to inhibit restenosis. Assessment of this process is crucial as successful re-endothelialisation has prognostic implications to the patient and would clearly impact and change his post-interventional follow-up and treatment. However, whereas the clinical assessment of endothelial recovery is primarily performed by estimating the extent of endothelium-dependent vasodilatation in response to acetylcholine or reactive-hyperaemia-induced shear stress or by gradual visualisation of endothelial cells by invasive imaging methods such as optical coherence tomography, non-invasive *in vivo* visualization of re-endothelialisation after PVI has not been successful until now^4,5^.

Recently, a molecular imaging probe that visualizes the expression of the transmembrane protein glutamate carboxypeptidase II (GCPII), also called prostate-specific membrane antigen (PSMA), has become available. Currently, the clinical application is tested in primary and recurrent prostate cancer^6^. GCPII is expressed in a variety of healthy (e.g., salivary glands, duodenal mucosa, subset of proximal renal tubular cells, and subpopulations of neuroendocrine cells in the colonic crypts) and neoplastic tissues^7^ (e.g. subtypes of transitional cell carcinoma, renal cell carcinoma, colon carcinoma, and peritumoural as well as endotumoural endothelial cells of neo-vasculature). GCPII seems to be a true molecular interface, integrating both extracellular and intracellular signals during angiogenesis. Especially the endothelial cell invasion seems to be dependent on GCPII activity since GCPII inhibition, knockdown, or deficiency decreases endothelial cell invasion *in vitro* and thereby abrogates angiogenesis^8^. Next to that, GCPII also plays a role in the neo-vasculature of physiologic regenerative and reparative conditions^9^.

Over the last years, GCPII has received increasing attention as a useful biomarker in the evaluation of prostate cancer patients with positron emission tomography (PET). The recent development of several ^68^Ga labelled GCPII inhibitors for PET imaging demonstrated a high specificity for GCPII expressing tumour cells *in vitro* and *in vivo*. The first human studies revealed a high specificity as well as a high detection rate in patients with prostate cancer using GCPII-inhibitor-PET^10–13^.

In this study, we evaluated the potential role of a newly developed ^18^F-labeled GCPII targeting tracer for *in vivo* molecular imaging of re-endothelialisation after PVI in the common carotid artery in a rat model and corroborated the results with immunohistochemistry of GCPII expression and polymerase chain reaction (PCR).

## Results

### PET

**Figure 1:**
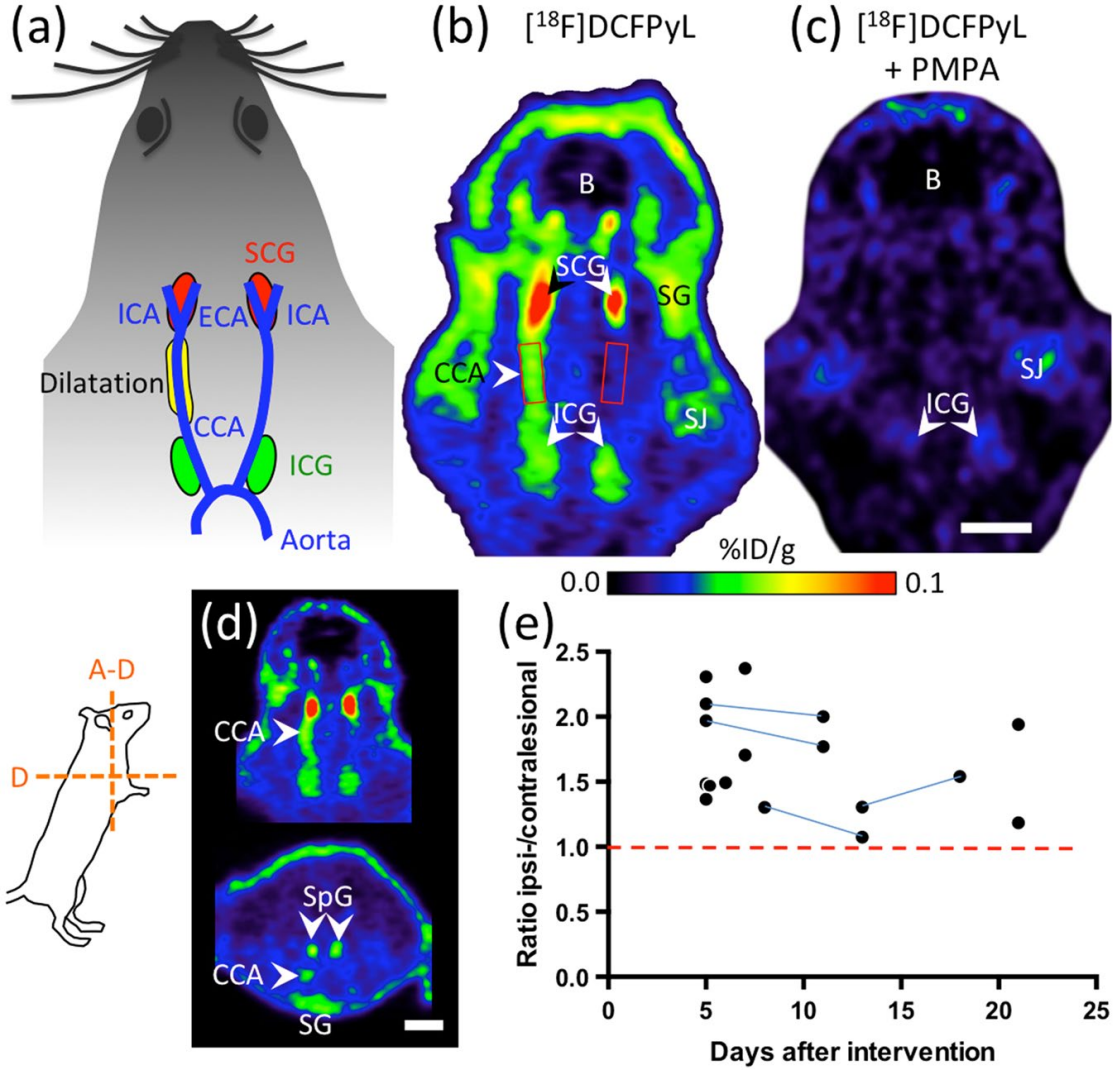
Increased GCPII-expression in the rat CCA after dilatation with a balloon catheter. (**a**) Schematic drawing of the dilatation site (yellow) and the cervical ganglia (red and green) used as landmarks. (**b**) [^18^F]DCFPyL-PET image five days post-op. The image was tilted (see insert, level a–d) so that the CCA was oriented in the horizontal plane. Red squares indicate position of the VOIs. (**c**) Blocking experiment with PMPA (23 mg/kg BW), injected together with [^18^F]DCFPyL. (**d**) Image of a different rat seven days post-op. in horizontal and transverse view. E: Plotting of VOI ratios (ipsi-/contralateral) over time after dilatation. Blue lines indicate animals that were measured twice. Abbreviations: B: brain; CCA: common carotid artery; ECA: external carotid artery; ICA: internal carotid artery; ICG: inferior cervical ganglion; SCG: superior cervical ganglion; SG: salivary gland; SJ: shoulder joint. SpG: Spinal ganglia. Scale bars: 1 cm.

Five to 21 days after balloon dilatation, [^18^F]DCFPyL binding was noticeable in the entire ipsilateral CCA, while binding in the contralateral CCA was weak or even absent (Fig. 1a,b). The mean ratio between ipsi- and contralateral CCA was 1.67 ± 0.39, which was significantly different from µ = 1.0 (p < 0.0001; one sample t-test). Comparison of the arterial tracer uptake between the ipsi- and contralateral CCA revealed significantly higher uptake values for the ipsilateral CCA (0.030 ± 0.026%ID/cm^3^ vs. 0.020 ± 0.021%ID/cm^3^, p = 0.0004; paired t-test). Blocking of GCPII binding sites with PMPA in two animals resulted in strong reduction of ipsilateral arterial tracer binding to 0.0008 and 0.0004%ID/ccm^3^, respectively (Fig. 1c).

### *Ex vivo* GCPII expression analysis

**Figure 2:**
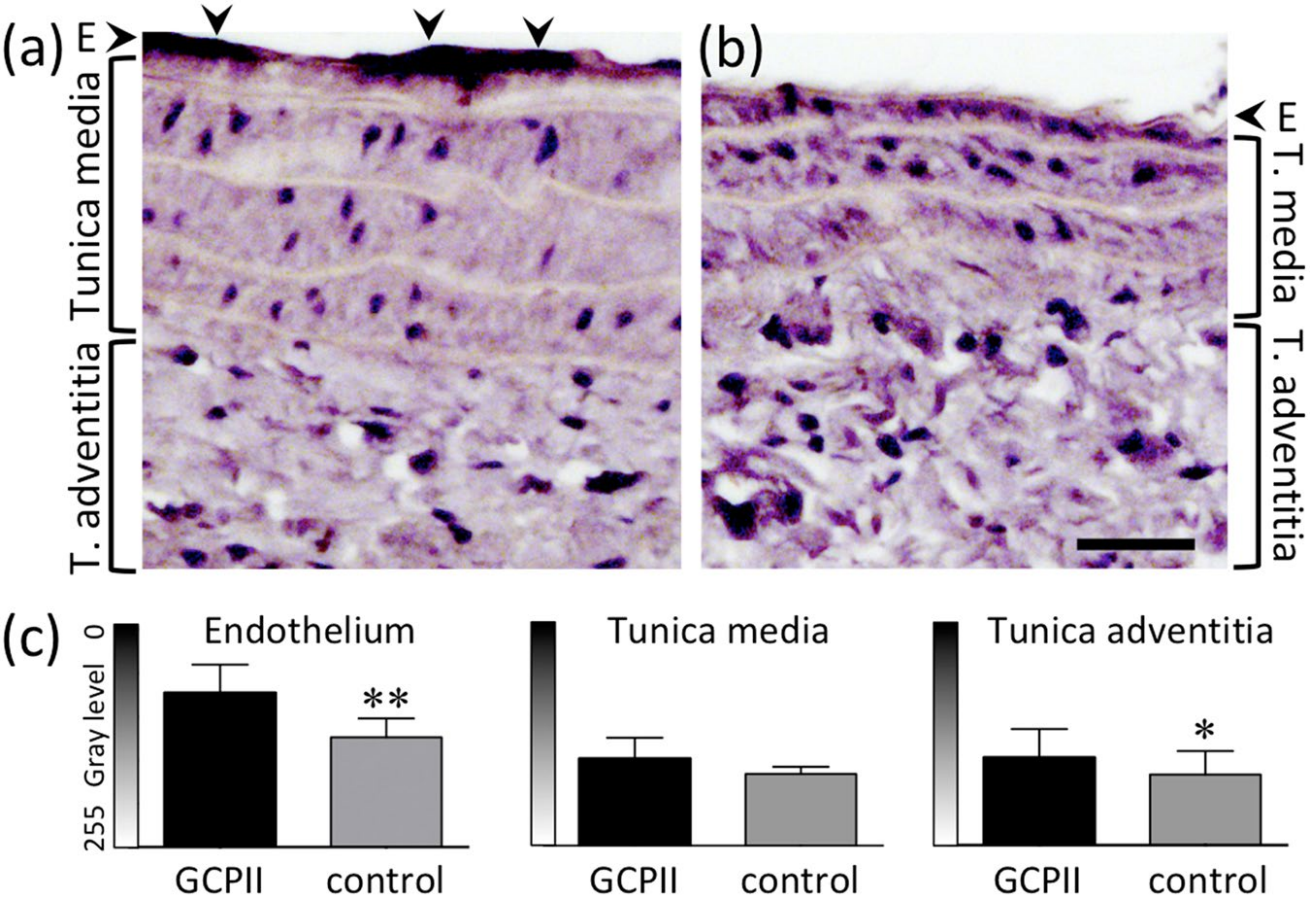
GCPII immunohistochemistry with HE counterstaining. (**a**) GCPII immunostaining of the dilated CCA. Arrowheads indicate immunopositive endothelial cells (E). (**b**) Negative control without primary antibody. (**c**) Mean gray values ± standard deviation. *p < 0.05, **p < 0.005. Scale bar: 30 µm.

**Figure 3:**
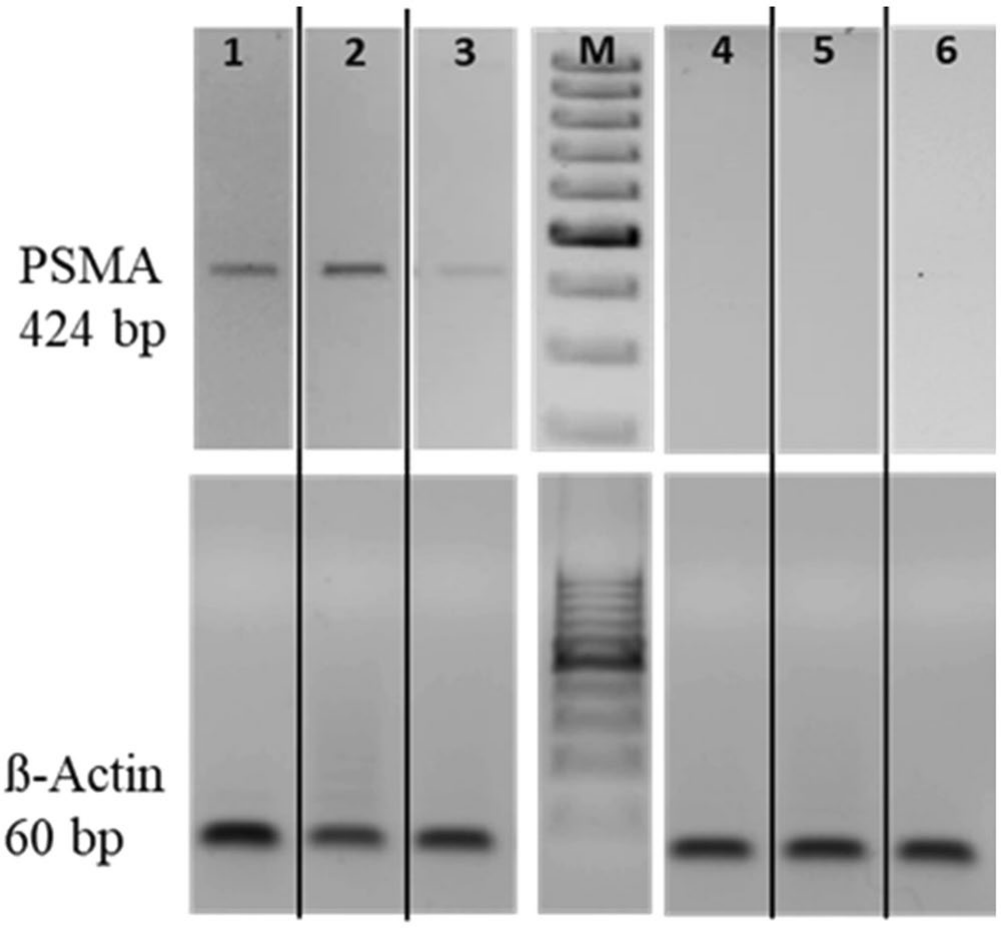
Comparative analysis of GCPII expression at mRNA level (cropped gels). Examples of PCR analysis of GCPII expression (product size 424 bp) in dilated CCAs (5, 7 and 21 days post balloon dilatation in lane 1, 2 and 3, respectively) and control CCAs (lanes 4–6). Corresponding β-Actin (product site 60 bp) served as a loading control (M, DNA ladder).

The vascular injury induced GCPII expression in the reparative endothelium of the ipsilateral CCA (Fig. 2a). GCPII staining was significantly more intense in endothelial cells of the dilated compared to the intact CCA (mean difference was 51 in a grayscale of 256 intensities, p = 0.0038, t-test; Fig. 2b,c). In the tunica adventitia, the external connective tissue layer, GCPII staining intensity was also higher in the dilated vessels, although this effect was not as pronounced as in the endothelial layer (mean difference: 20 gray levels, p = 0.0167). In the tunica media, the smooth muscle cell layer, GCPII staining intensity did not differ between dilated and intact CCA (mean difference: 18 gray levels, p = 0.1087). Corresponding to the immunohistological staining patterns, PCR tissue analysis revealed increased GCPII expression in the ipsilateral CCA, while no GCPII expression was detected in control CCAs (Fig. 3; supplementary Fig. 1).

## Methods

### Animals

**Figure 4:**
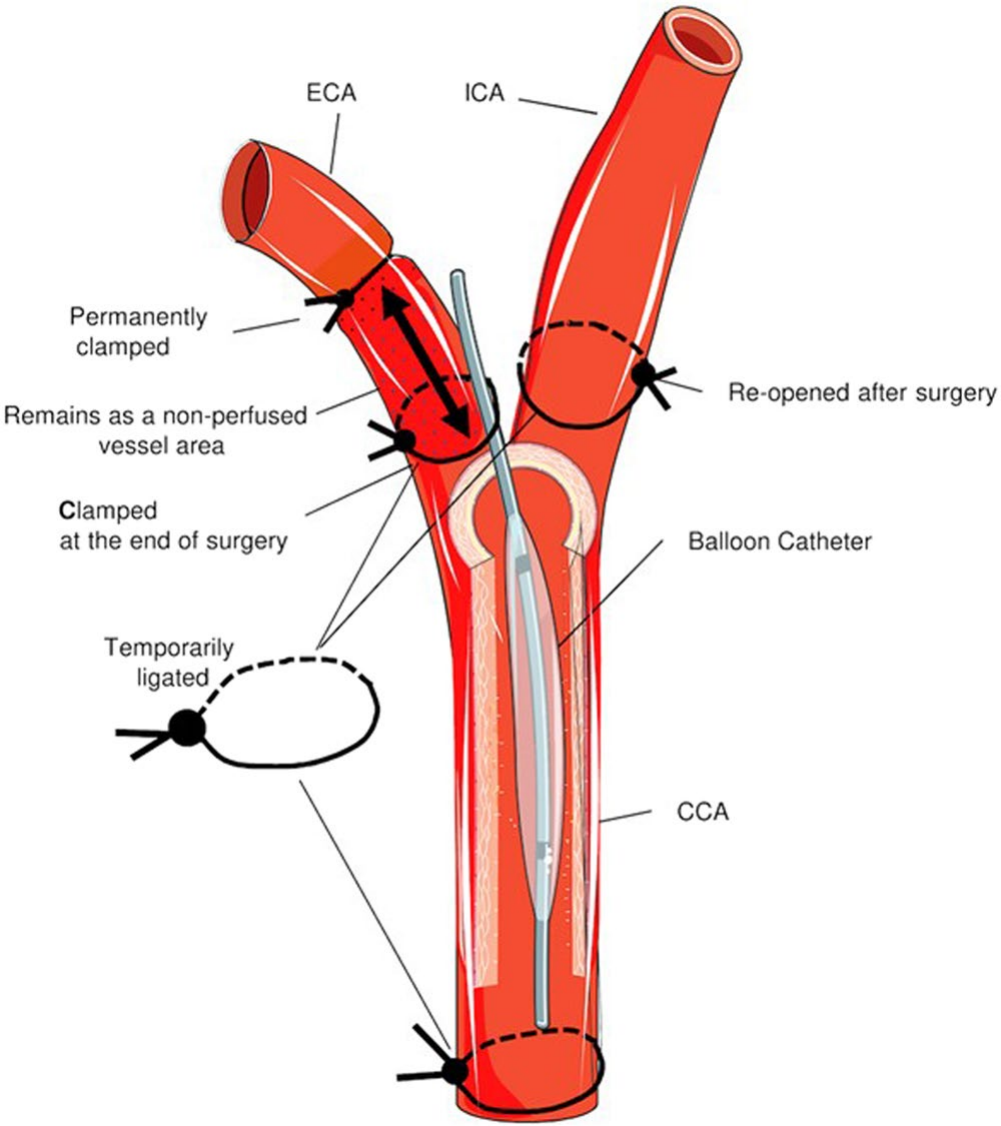
Schematic drawing of the surgical procedure. The balloon catheter is temporarily inserted via the external carotid artery (ECA) into the common carotid artery (CCA). The ECA remains clamped after surgery.

In total, 15 Sprague Dawley rats (male, 450–500 g) were included in this study. Angioplasty of the common carotid artery in the rats was performed under isoflurane anaesthesia. After midline neck incision, the left external carotid artery was ligated distally, and via transverse arteriotomy, a balloon dilatation catheter (2.0 × 8.0 mm, Mini Trek, Abbott Vascular) guided by a flexible angioplasty wire (diameter: 356 µm = 0.014 in) was advanced into the common carotid artery by 1 cm (Fig. 4). Complete and uniform endothelial denudation was achieved by expanding the balloon for 5 seconds with a pressure of 12 bar using an inflation syringe system (Medflator, Medex Supply, Monsey, NY). The muscular layers and skin incision were closed, and analgesics (buprenorphine 0.025 mg/kg BW) were administered until full recovery of the animal.

### Study protocol

PET image acquisition using the tracer [^18^F]DCFPyL was performed at different time points after angioplasty, from day 5 to day 21. Nine animals were measured once after the intervention, and four animals twice with an interval of 5–7 days between measurements. For blocking experiments, the GCPII inhibitor 2-(phosphonomethyl)pentane-1,5-dioic acid (PMPA; Tocris) was co-injected with the PET tracer at a concentration of 23 mg/kg BW in two animals.

Uptake analyses were carried out for the ipsilateral (dilated) and contralateral (untreated) common carotid artery (CCA). Animals were sacrificed after their last PET scan, and immunohistochemistry of both carotid arteries was performed using a GCPII-specific antibody.

### Synthesis and radiolabelling of the GCPII targeting radiotracer [^18^F]DCFPyL

[^18^F]Fluoride was produced via the ^18^O (p, n)^18^F reaction by bombardment of enriched [^18^O] water with 16.5 MeV protons using a MC16 cyclotron (Scanditronix, Uppsala, Sweden) at the Max Planck Institute for Metabolism Research in Cologne. The synthesis of [^18^F]DCFPyL was carried out as follows^14^. A solution of 2,3,5,6-Tetrafluorophenyl-6-[^18^F]fluoronicotinate ([^18^F]F-Py-TFP) (0.5–30 GBq) in EtOH (500 µL), prepared according to the procedure of Malik *et al*., was added to 2-[3-(1,3-dicarboxypropyl)-ureido]pentanedioic acid tris-tert-butyl ester (2.5 mg) and the reaction mixture was stirred for 10 min at 40 °C^15,16^. Thereafter, 10 M HCl (1 mL) was added and the mixture was stirred for 10 min at 100 °C. Volatiles were removed under reduced pressure and the residue was taken up in 0.38% H_3_PO_4_ (pH 2) and purified by semipreparative HPLC to give [^18^F]DCFPyL in reasonable radiochemical yields of 8–12% and in high radiochemical purity (99.8%). The final product was formulated in a PBS solution (pH 4–6). The specific activity of [^18^F]DCFPyL amounted to 65–78 GBq/μmol. Semi-preparative HPLC was performed with a Chromolith SpeedROD® column of 50 × 4.6 mm (Merck Millipore, Darmstadt, Germany), 5% EtOH in 0.38% H_3_PO_4_ (pH 2) as eluent and a flow rate of 3.0 mL/min. Quality control was conducted under the same conditions as for the purification with tR = 2.2 min.

### PET acquisition

13–80 MBq of [^18^F]DCFPyL in isotonic saline (200 µL) were injected into the lateral tail vein of the rats. One hour later, rats were set under anaesthesia with isoflurane and placed in a small animal PET scanner (Inveon, Siemens, Knoxville, USA) under continuous ECG and temperature monitoring and imaged for 60 minutes. CT images were acquired on a Philips Gemini TF16 PET/CT (Philips Medical Systems, Best, The Netherlands).

### PET image analysis

PET images were reconstructed with the help of a 3D-OSEM procedure yielding a matrix of 128 × 128 × 159 voxels with sizes of 0.78 × 0.78 × 0.80 mm^3^. Using the software VINCI^17^, all PET images were Gauss-filtered with a kernel of 1.0 mm FWHM and co-registered to the CT images. For VOI analysis, images were rotated so that superior and inferior cervical ganglia, which were labelled in all preparations, appeared in the same horizontal plane on both sides of the body. Because the CCA stretches between the two ganglia, this procedure aligns left and right CCA with the horizontal plane as well, and the damaged vessel can be easily recognized (Fig. 1). Two cylindrical volumes of interest (VOIs) were drawn with a diameter of 2.9 mm and a length of 7.3 mm, comprising 101 voxels each. The VOIs were positioned over the part of the vessel where the catheter was inserted, and over its contralateral counterpart, respectively (Fig. 2a). If the unaffected contralateral CCA was not visible at all, the contralateral VOI position mirrored that of the ipsilateral VOI. Mean VOI values in % injected dose per cm³ (%ID/ccm) tissue were extracted, and the ratio between ipsilateral (=dilated) and contralateral (=untreated) side was calculated.

All continuous variables are expressed as mean ± standard deviation and categorical data as absolute numbers and percentages. A Pearson correlation analysis was performed between the ratio of ipsi- and contralateral CCA [^18^F]DCFPyL uptake and post-operative day. Since the correlation was not significant (R = −0.28; p = 0.2699), all measurements were pooled and a paired t-test was used to compare ipsi- and contralateral CCA.

### Immunohistology

Consecutive formalin-fixed, paraffin-embedded longitudinal tissue sections (2 µm thick) were dewaxed in xylene and rehydrated through graded concentrations of ethanol to distilled water. Sections were then immersed in 10 mmol/L citrate buffer (pH 6.0) and processed for antigen retrieval in the microwave for 5 min at 600 W. For blocking of endogenous peroxidase activity the sections were treated with 0.3% H_2_O_2_ solution for 15 min at RT. Unspecific antibody binding was inhibited by incubation in 3% BSA-TBST solution for 30 min at RT. For staining, sections were incubated with the anti-PSMA (=anti-GCPII) antibody (1:200 dilution in TBST, clone YPSMA-1, abcam) over night at 4 °C. Subsequently, the sections were washed tree times with TBST and exposed for 60 min to peroxidase-linked anti-mouse immunoglobulin antibody (1:500 diluted in TBST, Cell Signaling Technology). Colour development used diaminobenzidine substrate and sections were counterstained with haematoxylin/eosin (HE). Digital photographs of the stained arteries were analysed with ImageJ 1.51m9 (NIH, USA). For analysis of the endothelial cell layer, five short lines per section were drawn perpendicular to the luminal surface, and profile plots (gray value against distance) were generated. The minimum gray value corresponding with the darkest pixel in the endothelial layer was extracted from each plot. For the tunica media and adventitia, five rectangular regions of interest (ROIs) were drawn per section, and the ROI mean values were retrieved. Mean values for each animal from the dilated artery (GCPII staining versus control staining without primary antibody) were compared using a paired t-test.

### PCR validation of GCPII expression

Total RNA was extracted from formalin-fixed, paraffin-embedded tissue sections using a Phenol-chloroform method. Reverse transcription was carried out using Advantage RT-for-PCR kit (Clontech, Mountain View, California, USA) (50 ng RNA/sample). RNA and cDNA were quantified by using a BioPhotometer (Eppendorf, Germany). For PCR analysis (0.5 µg cDNA/sample) gene-specific primers were used at 10 pmol per reaction: GCPII forward primer 5′-TGCAGGGCTGATAAGCGAGGCATT-3′; GCPII reverse primer 5′-TGGGATTGAATTTGCTTTGCAAGCTG-3′; Actin-β forward primer 5′-CCCAGCACAATGAAGATCAA-3′, Actin-β reverse primer 5′-GATCCACACGGAGTACTTG-3′. PCR was performed using Advantage® cDNA PCR kit (Clontech) for 35 cycles. PCR products were resolved on a 1.2% agarose gel, stained with ethidium bromid, and bands were detected using the ImageQuant LAS 4010 camera system (GE Healthcare).

### Ethical statement

Animal experiments were performed in accordance with the German legislation governing animal studies following the ‘Guide for the care and use of Laboratory Animals’ (NIH publication, 8th edition, 2011) and the Directive 2010/63/EU on the protection of animals used for scientific purposes (Official Journal of the European Union, 2010). Official permission was granted from the governmental animal care and use office (LANUV Nordrhein-Westfalen, Recklinghausen, Germany).

The datasets generated during and/or analysed during the current study are available from the corresponding author on reasonable request.

## Discussion

Using microPET and PCR, we found a significantly increased GCPII expression in the ipsilateral CCA compared to the contralateral, non-treated CCA between day 5 and 21 after balloon denudation. Immunohistochemical stainings showed that the endothelial cell layer was the main source of elevated GCPII, while approx. 28% of the signal arose in the tunica adventitia. Although endothelial cells form a very thin layer (a few micrometers at most), it is possible to visualize purely endothelial targets with PET, as has been shown for endothelial HSP-60 expression in the rabbit aorta^18^. Because GCPII is highly expressed in neo-vasculature of reparative and regenerative tissues^9^, our findings most likely reflect an elevated re-endothelialisation process after endothelial denudation rather than the injury *per se*. Increased GCPII expression in the adventitia may indicate formation of neo-vasculature^19^ and/or the recruitment of resident progenitor cells to form new endothelial cells^20^. Thus, GCPII expression most likely represents the healing processes, taking place in different layers of the dilated vessel.

Arterial injury is an unavoidable consequence of all interventional vascular procedures, which is followed by a cascade of cellular and molecular events resulting in an acute damage of the endothelial layer of the arterial vessel wall^21^. Disruption of the normal endothelial structure or function is strongly associated with the pathogenesis of atherosclerosis. Furthermore, it also leads to early, late, and very late thrombotic events and re-stenosis burden that are well-known to occur after angioplasty and stenting^2^. Based on these pathophysiological processes, it becomes obvious that maintaining or re-establishing a competent and fully intact endothelium in the treated vessel is crucial for the long-term health of the respective vessel wall. Consequently, the process of endothelial restoration has significant prognostic and therapeutic implications for patients undergoing PVI^2^. Current therapeutic concepts are therefore focused on the acceleration of re-endothelialisation after PVI, at the same time aiming to inhibit restenosis^2^. The introduction of drug-eluting stents several years ago has reduced the rate of early re-stenosis, however late re-stenosis or neo-atherosclerosis is a common side effect, requiring a more pronounced anti-platelet therapy. The problem of late re-stenosis is most likely due to a still incompetent and not fully restored endothelium with respect to its integrity and function^2^. In this context, assessing the process of re-endothelialisation *in vivo* and, consequently, healing of the injured vessel wall is mandatory and much-needed. However, despite remarkable advances in imaging techniques, imaging of the endothelial cell structure and integrity is challenging and still cannot currently be assessed adequately *in vivo*. Up to now, the assessment of endothelial function in the clinical situation has primarily been performed by estimating the extent of endothelium-dependent vasodilatation in response to acetylcholine or reactive-hyperaemia-induced shear stress^4,5^. Using optical coherence tomography it was demonstrated that the abnormal vasoconstriction in response to acetylcholine three months after implantation of a drug-eluting stent is associated with the presence of uncovered struts^22^. Optical coherence tomography is an *in vivo* technique that provides high-resolution images. The technique enables identification of the detailed morphology of coronary plaques, including the thickness of the fibrous cap and the accumulation of macrophages. However, the recognition of the structure of the endothelium in native coronary arteries remains inconclusive^23^.

Non-invasive molecular imaging of GCPII expression after PVI seems to have the potential to overcome this shortcoming as GCPII plays a significant role in angiogenesis, mainly with regard to endothelial cell invasion, as this process seems to be GCPII-dependent^8^. GCPII also plays a significant role in the neo-vasculature of physiologically regenerative and reparative conditions. It is assumed that the folate hydrolase activity of GCPII facilitates angiogenesis by increasing local availability of folic acid^9^. The increased local availability of folic acid in endothelial cells seems to lead to an increase in the amount of tetrahydrobiopterin. Tetrahydrobiopterin in turn facilitates the VEGF-mediated production of nitric oxide by endothelial nitric oxide synthase^9^. Nitric oxide is known to exhibit several vasoprotective effects, for instance vascular relaxation and endothelial regeneration. Furthermore, it inhibits platelet aggregation and blood coagulation^24^.

The visual and the semi-quantitative analysis of the tracer uptake in the CCAs as well as the results of the immunohistochemistry indicate a higher tracer uptake in the injured ipsilateral CCA compared to the contralateral CCA. Therefore, we are confident that this is a very promising proof-of-concept finding and the next step will be the translation of this application. This could be done in a rather short time frame since the tracer itself is already applied in a clinical setting.

For the first time, we provide evidence that GCPII is a reliable *in vivo* marker of re-endothelialisation after PVI and that GCPII-PET is the first non-invasive *in vivo* molecular imaging technique that can demonstrate and quantify re-endothelialisation. As a further perspective, well-powered studies in patients undergoing PVI need to confirm the potential of GCPII-PET for imaging the crucial process of arterial re-endothelialisation after injuring the vessel wall.

## Electronic supplementary material

Supplementary figure 1
