# Supplementary material for: In vivo Molecular Imaging of Glutamate Carboxypeptidase II Expression in Re-endothelialisation after Percutaneous Balloon Denudation in a Rat Model
Source: Sci Rep. 2018 May 9;8:7411. doi: 10.1038/s41598-018-25863-1 (PMC5943322; doi:10.1038/s41598-018-25863-1)
Supplement: Supplementary file 1 — Supplementary figure 1 [file 41598_2018_25863_MOESM1_ESM.docx]

**- Supplemental Information –**

***In vivo* Molecular Imaging of Glutamate Carboxypeptidase II**

**Expression in Re-endothelialisation after Percutaneous Balloon**

**Denudation in a Rat Model**

Heike Endepols^1,2,+^, Felix M. Mottaghy^3,4,+,*^, Sakine Simsekyilmaz^5^, Jan Bucerius^3,4,6^, Felix Vogt^7^, Oliver Winz^3^, Raphael Richarz^1^, Philipp Krapf^1,9^, Bernd Neumaier^1,8,9^, Boris D.

Zlatopolskiy^1,3,+^, Agnieszka Morgenroth^3,+^

^1^ Institute of Radiochemistry and Experimental Molecular Imaging (IREMB), University

Hospital of Cologne, 50937 Cologne; Germany

^2^ Department of Nuclear Medicine, University Hospital of Cologne, 50937 Cologne,

Germany

^3^ Department of Nuclear Medicine, University Hospital, RWTH Aachen, 52074 Aachen;

Germany

^4^ Department of Nuclear Medicine, Maastricht University Medical Centre (MUMC+), 6229

HX Maastricht, The Netherlands

^5^ Institute for Molecular Cardiovascular Research, University Hospital, RWTH Aachen,

52074 Aachen; Germany

^6^ Cardiovascular Research Institute Maastricht (CARIM), Maastricht University Medical

Centre (MUMC+), 6229 HX Maastricht, the Netherlands

^7^ Department of Cardiology, Pneumology, Angiology, and Internal Intensive Care Medicine,

University Hospital, RWTH Aachen, 52074 Aachen; Germany

^8^ Max Planck Institute for Metabolism Research, 50931 Cologne; Germany

^9^ Institute for Neuroscience and Medicine (INM-5), Nuclear Chemistry, Research Centre

Jülich, 52425 Jülich; Germany

* Corresponding author: fmottaghy@ukaachen.de

^+^ These authors contributed equally to this work.


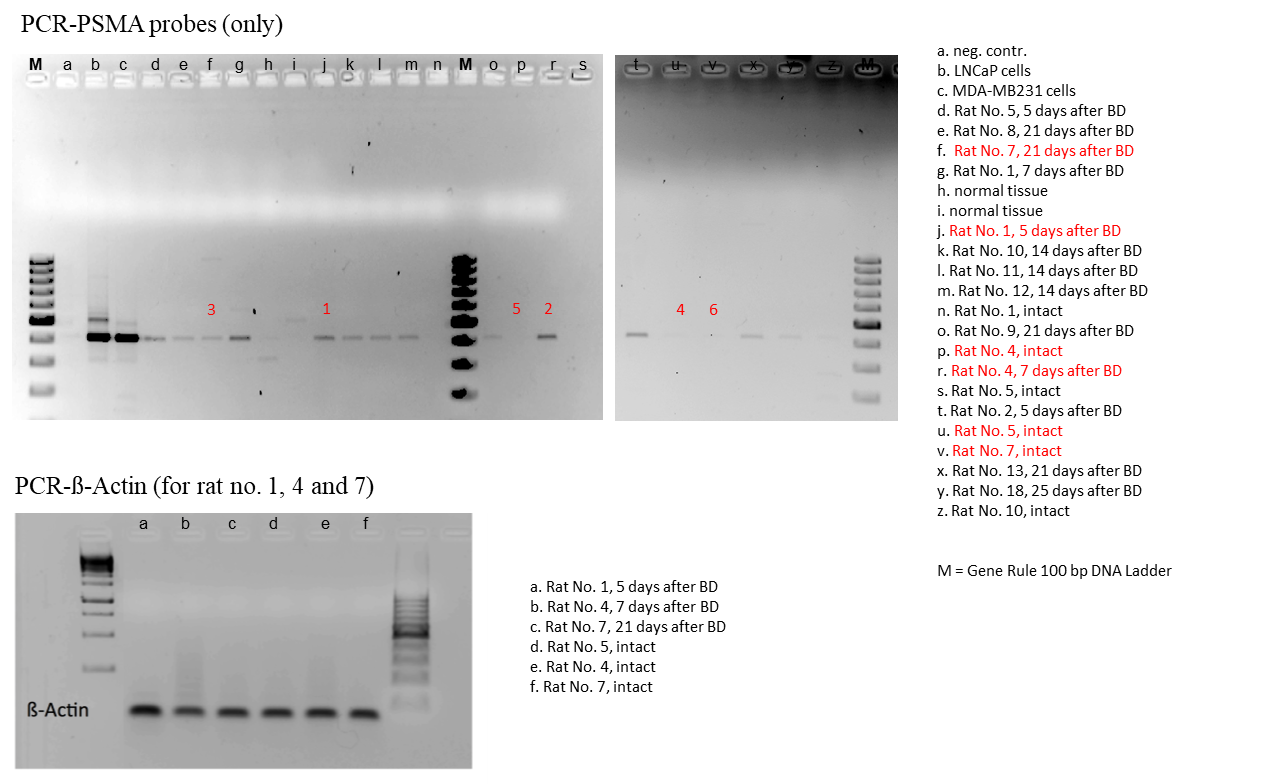


Supplemental figure 1:

PCR analysis of PSMA and β-actin expression, full-length gels. The paired PSMA analysis (vessels after balloon dilatation and ipsi-lateral intact vessels) with corresponding ß-actin analysis presented in the publication are marked in red.
